# Clarifying the role of three-dimensional transvaginal sonography in reproductive medicine: an evidenced-based appraisal

**DOI:** 10.1186/1743-1050-2-10

**Published:** 2005-08-11

**Authors:** Nick Raine-Fenning, Arthur C Fleischer

**Affiliations:** 1 Obstetrics & Gynaecology, Queen's Medical Centre, University Hospital NHS Trust, NURTURE, B Floor, East Block, Nottingham, NG 7 2UH UK; 2 Department of Radiology and Radiological Sciences, Vanderbilt University Medical Center, 1116 21^st ^Avenue, South, Nashville, TN 37232-2675 USA; 3 Department of Obstetrics and Gynecology, Vanderbilt University Medical Center, 1116 21^st ^Avenue, South, Nashville, TN 37232-2675 USA

## Abstract

This overview describes and illustrates the clinical applications of three-dimensional transvaginal sonography in reproductive medicine. Its main applications include assessment of uterine anomalies, intrauterine pathology, tubal patency, polycystic ovaries, ovarian follicular monitoring and endometrial receptivity. It is also useful for detailed evaluation of failed and/or ectopic pregnancy. Three-dimensional color Doppler sonography provides enhanced depiction of uterine, endometrial, and ovarian vascularity.

## Background

**Figure 1:**
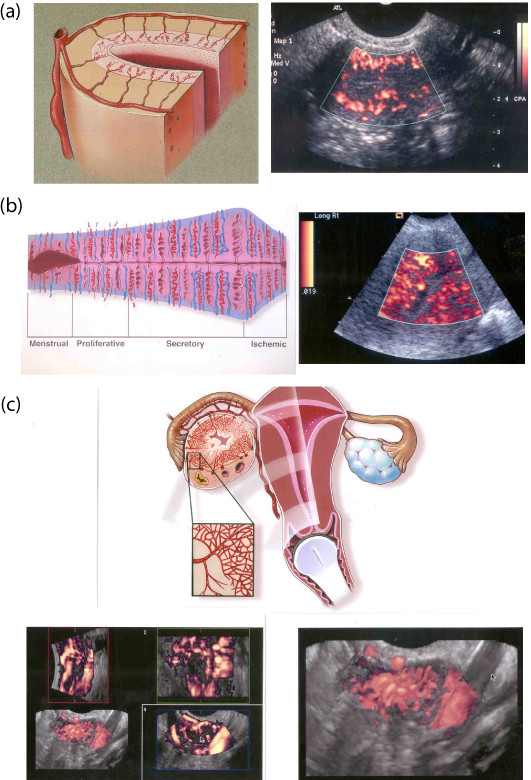
Two-dimensional vs three-dimensional transvaginal color Doppler sonography. (a) Diagram (left) and two-dimensional TV-CDS (right) showing arcuate, radial, and spiral vessels in follicular phase. (b) Diagram (left) are two-dimensional TV-CDS (right) showing changes in endometrial vascularity throughout the menstral cycle. During the luteal phase, several spiral vessels are detected. (c) Diagram (top) and multiplanar images (left) and magnified 3D TV-CDS (right) of corpus luteum. The multiplanar image shows the vascular "wreath" surrounding the functioning corpus luteum in the longitudinal (top left), short (axial) (top right) and coronal (bottom right) planes. The combined gray scale and 3D TV-CDS image (both left) which is also magnified and shown as the right depicts the numerous vessels surrounding this corpus luteum.

Conventional sonography provides two-dimensional views of three-dimensional structures that an experienced ultrasonographer has to dynamically examine in order to create their own three-dimensional impression of the object of interest [1]. In contrast, three-dimensional sonography allows the simultaneous assessment of individual sectional planes, which dependent upon the particular field of interest may be examined in one of several different viewing modalities to maximise the information available and improve spatial awareness (Fig. 1) [2,3]. Uniquely, three-dimensional sonography allows demonstration of the coronal plane perpendicular to the transducer face facilitating the identification of surface irregularities which can then be accounted for during volume measurement [4]. The digital technology central to its development also means that three-dimensional imaging lends itself to telemedicine, as it allows the storage of large datasets without loss of information that may be subsequently analysed off-line and reappraised by experts in a 'virtual real-time consultation' [5]. Two-dimensional color Doppler sonography provides a subjective estimation of uterine and ovarian vascularity. It is limited, however, by providing flow depiction in a single plane as opposed to the sample volume as obtained by three-dimensional imaging (Fig. 1). Advocates of three-dimensional sonography [6] suggest that these features offer the user the following advantages in comparison to two-dimensional sonography:

• accurate measurement of organ dimensions and volumes

• improved anatomic and blood flow information

• improved assessment of complex anatomic anomalies

• a better specificity in regard to the confirmation of normality

• standardisation of the sonographic examination procedure

• reduced scanning times with cost-effective use of equipment and sonographer time

• telemedicine and tertiary consultation

This review will critically appraise the evidence to see if the potential advantages of three-dimension over two-dimension may be substantiated in the context of reproductive medicine and specifically ask if they actually lead to an improved diagnostic capability. To begin it is first necessary to outline how three-dimensional sonography may be used to quantify volume and blood flow as these measurements form the basis of many studies.

### Three-dimensional Data Analysis

**Figure 2:**
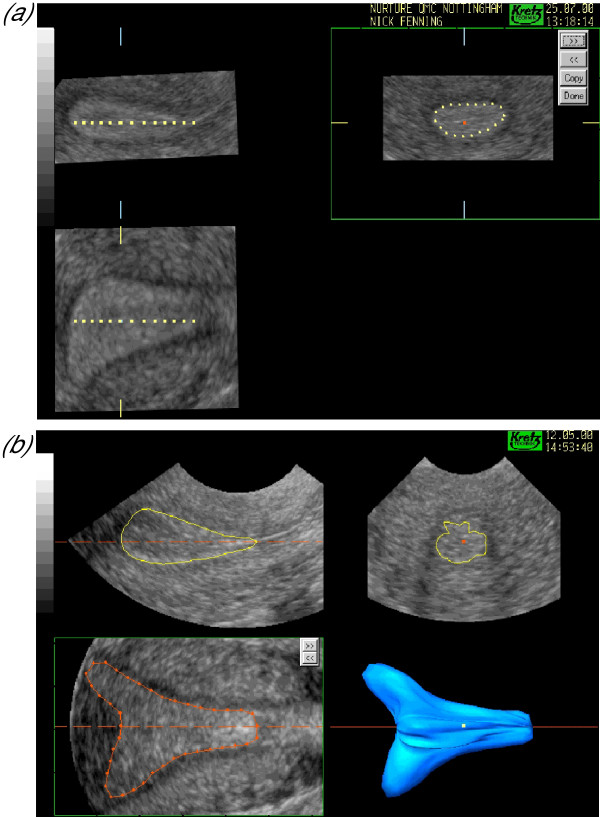
***The techniques of volume calculation***. Both figures show the typical multiplanar display of a three-dimensional sonographic dataset of the uterus with the three mutually related orthogonal planes at 90-degrees to one another. The upper left image in both displays represents the longitudinal plane (the A plane), the upper right image the transverse plane (the B plane) and the lower left image the coronal plane (the C plane). Volume calculation can be conducted in either the B or C plane. (a) shows the conventional technique of volume calculation in which a series of 'slices' are taken through the volume of interest whilst the contour is outlined in another plane (the transverse plane in this case). The distance between consecutive slices can be varied according to the degree of change in the surface contour and increased for more complex structures. (b) shows the rotational technique of volume calculation in which the dataset is rotated through 180° about a central axis defined by the application of two callipers. The number of planes available for volume calculation are determined by the rotation step shown in the lower left of the image. Here the 30-degree rotation step has been used and the contour outlined in the coronal or C plane using the manual mode. The resultant three-dimensional model is shown in the lower right of the image.

**Figure 3:**
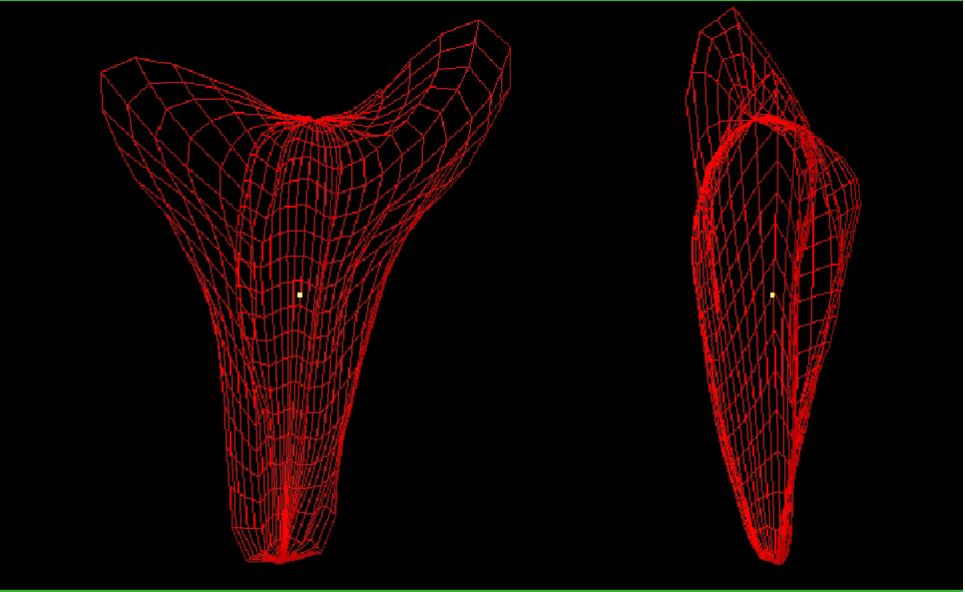
Three-dimensional 'wire' models of the endometrium. When VOCAL is used to calculate volume a three-dimensional model is generated that may be rotated and examined in its natural orientation. In this model the coronal view is seen on the left clearly demonstrates the fundal defect of an arcuate uterus whilst the actual anteflexion of the uterus may be appreciated in the lateral view on the right.

**Figure 4:**
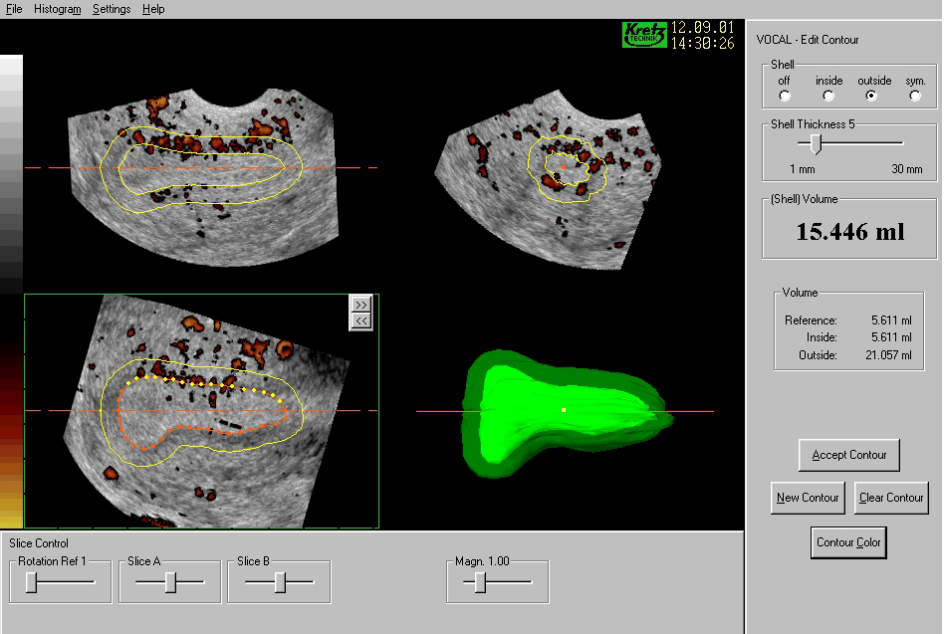
***Three-dimensional power Doppler angiography of the uterine blood supply***. A three-dimensional dataset containing power Doppler information has been acquired from the uterus. VOCAL has then been used to define the myometrial-endometrial border and to apply a shell 5 mm outside of that contour to define the sub-endometrium, which can be clearly seen to be more vascular than the endometrium itself.

**Figure 5:**
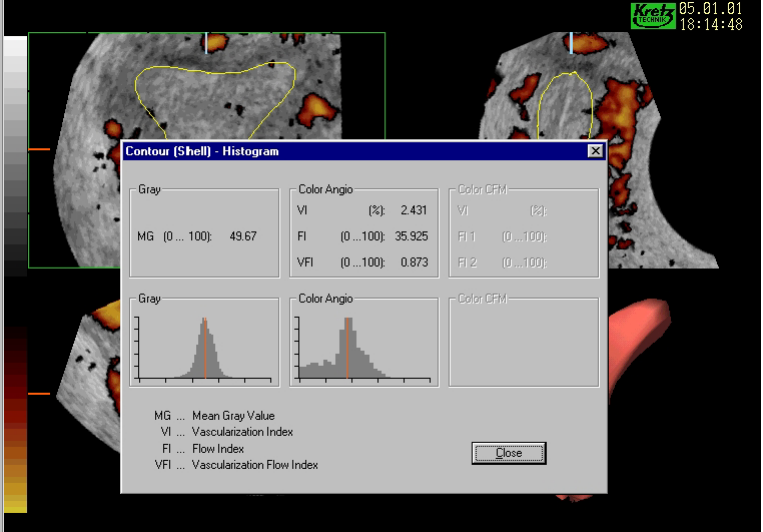
***The 'Histogram'*. **The three indices of vascularity have been calculated for the endometrial model together with the mean grey value. The Vascularisation Index (VI) reflects the degree of power Doppler information within the model and is considered as a percentage therefore whilst the Flow Index (FI) and Vascularisation Flow Index (VFI) include information on the mean power Doppler signal intensity referenced against a scale from zero to one hundred to indicate the minimal and maximum range accordingly.

**Figure 6:**
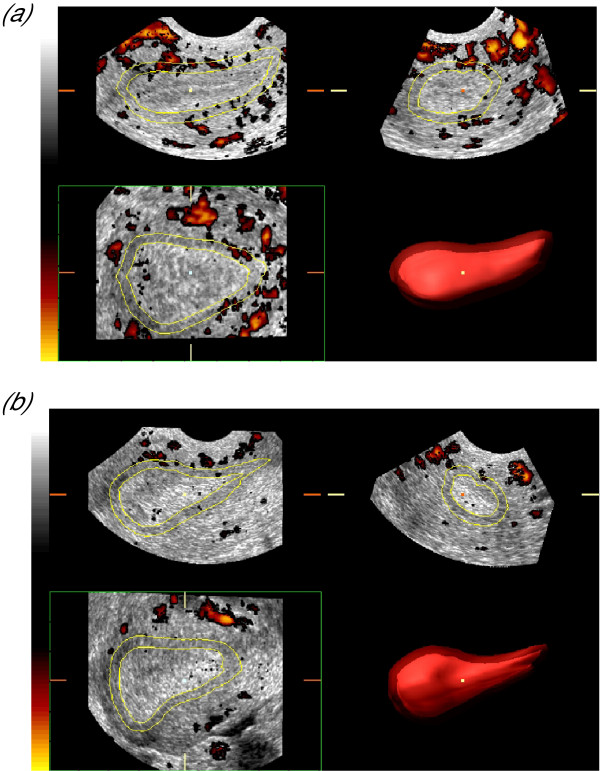
Intra-subject variation in endometrial blood flow. The degree of power Doppler information seen within Figure 6a is clearly superior to that seen in Figure 6b and this difference is quantifiable through the 'histogram' facility. These images also serve to demonstrate how vascularity is independent of morphometry and varies throughout different phases of the menstrual cycle as the less vascular homogenous endometrium characteristic of the luteal phase in Figure 6b is of greater volume than that in Figure 6a, which is the same endometrium at the end of follicular phase.

When one thinks of three-dimensional imaging in terms of its measurement capability the most obvious parameter considered is that of volume. Whilst volume may be estimated from measurements made with conventional two-dimensional sonography, such measurements use various formulae based upon certain geometric assumptions [7]. Volume estimation based on three-dimensional sonography still involves a degree of geometric assumption, as data are reconstructed based upon their most probable position within a Cartesian grid system, but utilises much more information. There are two basic methods employed to calculate volume from a three-dimensional dataset: the conventional 'full planar' or 'contour' method (Fig. 2a) and the more recently introduced 'rotational' method possible through the VOCAL-imaging program (Virtual Organ Computer-aided AnaLysis™) (Fig. 2b) which also generates a three-dimensional model of the object of interest (Fig. 3). Volume calculation by either of these techniques has proven highly reliable and valid both *in vitro *and *in vivo *[8-15]. Both techniques involve the manual delineation of the object of interest in the multiplanar display that shows the three perpendicular planes characteristic of three-dimensional sonography but there are other advantages to the 'rotational' technique in that it facilitates assessment of blood flow in a novel manner through the quantification of the power Doppler signal both within the defined volume of interest and also within the surrounding tissue through the application of a shell parallel to the originally defined surface contour (Fig. 4). Three indices of vascularity are calculated: the Vascularisation Index (VI) reflects the ratio of power Doppler information within the total dataset relative to both colour and grey information, the Flow Index (FI) represents the mean power Doppler signal intensity and the Vascularisation Flow Index represents a combination of the two (Fig. 5) [16]. The exact relationship of these indices to true flow and vascularity *in vivo *remains to be established but they have been shown to vary both within an individual and between different subjects suggesting they could have a valuable role in identifying and categorising differences between patient groups (Fig. 6). Importantly, the indices may be calculated in a reproducible manner between observers [17] following the three-dimensional acquisition of power Doppler data which itself has also been shown to be reliable [18].

## Clinical applications of three-dimensional sonography

Having established the key features of three-dimensional sonography in terms of its measurement ability and improved spatial awareness let us now examine how these have been applied in the diagnosis of subfertility and subsequent monitoring of treatment.

### Investigation of subfertility

Three-dimensional ultrasound has been used to diagnose uterine anomalies, assess tubal patency and to exclude intrauterine and ovarian pathology.

#### • Uterine anomalies

**Figure 7:**
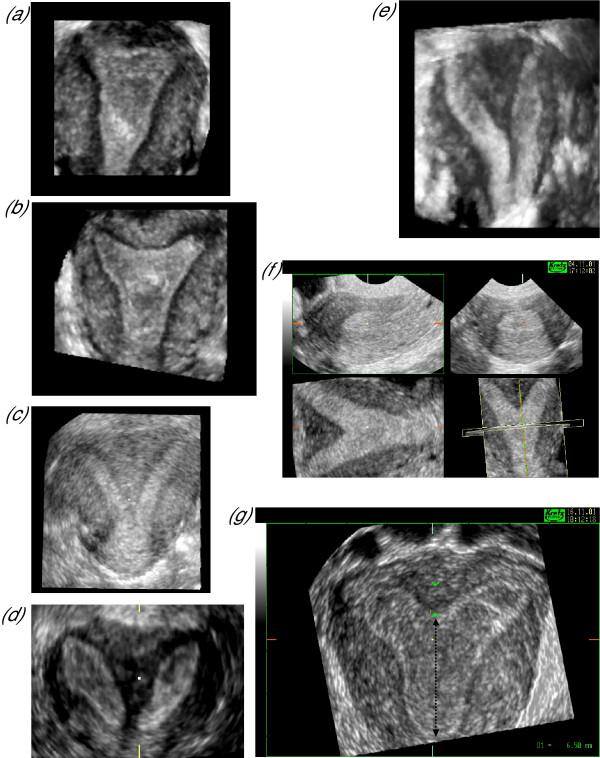
***Uterine anomalies***. Three-dimensional sonography has become the 'gold standard' investigation for the diagnosis and exclusion of congenital uterine anomalies. Its extremely high sensitivity and specificity relate to its ability to demonstrate the plane coronal perpendicular to the transducer face and in doing so allow visualisation of the fundal contour and comparison of the myometrium with the endometrium throughout the uterine length. Figure 7a shows normal cornna with straight contours at the upper aspect of the cavity in contrast to the characteristic concave contour seen in arcuate uteri (Fig. 7b) and the deeper contour of various length seen in sub-septate uteri (Figs. 7c, 7d & 7e). Any indentation of the fundal contour may also be appreciated in the coronal plane as seen in the multiplanar display in Figure 7f. This uterus had been considered normal with conventional ultrasound, which only provides the longitudinal and transverse views seen in the upper two images, but laparoscopy had demonstrated a bulky uterus with a possible fundal defect and a follow-up three-dimensional ultrasound confirmed the presence of a significant septum. The size of the septal defect can be measured as shown in Figure 7g but may be less important than the remaining length of the cavity shown as a bold dashed line.

Congenital uterine anomalies are associated with an increased risk of repeated first and second trimester miscarriage and preterm delivery [19]. A meta-analysis of published retrospective data comparing pregnancy outcome before and after hysteroscopic septoplasty indicated a marked improvement after surgery, which itself has minimal postoperative sequelae [20]. Accurate and reliable diagnosis is important therefore as it allows the identification of patients at risk of these complications and timely surgical intervention. This is undoubtedly the area where three-dimensional sonography has contributed the most and has become the investigation of choice in units where available. This reflects its ability to demonstrate both the endometrial cavity and the myometrium simultaneously in the coronal plane as shown by Jurkovic et al. in their pioneering study of 61 patients with a history of recurrent miscarriage or infertility (Figs. 7) [21]. Hysterosalpingography had shown the presence of a normal uterus in 44 (72.1%) patients, an arcuate uterus in nine (14.8%) and a major fusion defect in three cases (4.9%). Whilst two-dimensional sonography was associated with five false-positive diagnoses of arcuate uteri and three of major uterine anomalies, three-dimensional sonography agreed with hysterosalpingography in all of these cases. Shortly afterwards Raga et al. and Wu et al. both reported similarly favourable results suggesting three-dimensional sonography offered a 100% specificity for the exclusion of uterine anomalies and was able to differentiate between the different anomalies [22,23].

Three-dimensional sonography has since been used to determine the prevalence of uterine anomalies in various patient groups and to characterise outcome on the basis of the anomaly. As many as 24% of women with recurrent pregnancy loss may have uterine anomalies [14] which is roughly four times that seen in low-risk women where the prevalence is in the order of 5 to 6% [25,26]. In terms of the type of anomaly a similar distribution is seen between different groups with arcuate uteri being the most common, followed by subseptate then bicornuate uteri with the more complex anomalies such as uterus didelphys and single uterine horns the least prevalent. Women with a subseptate uterus have a significantly higher proportion of first-trimester loss and women with an arcuate uterus a significantly greater proportion of second-trimester loss (p < 0.01) and preterm labor (p < 0.01) compared to women with a normal uterus [24]. Another important finding derived from these three-dimensional studies has been that outcome is related not only to the degree of defect but also to the remaining cavity length which is significantly shorter in both arcuate and subseptate uteri in women with recurrent miscarriage [26]. These measurement techniques and classifications used allow comparison between these studies as a degree of standardisation has been used, based on measurement of fundal distortion from the mid-point of an imaginary horizontal line joining the upper aspects of the cornuae to the upper aspect of the uterine cavity, that has been shown to be reliable between observers examining stored three-dimensional datasets (Fig. 7f) [27].

If we revisit the advantages proposed at the start of the review we can see that the majority are already satisfied in respect to uterine anomalies. Three-dimensional sonography offers a reliable and standardised tool to diagnose, differentiate and quantify uterine anomalies. Three-dimensional sonography has significantly added to our understanding of uterine anomalies qualifying their effect on reproductive outcome and thereby helping the clinician counsel patients accordingly and confidently.

#### • Intrauterine pathology

**Figure 8:**
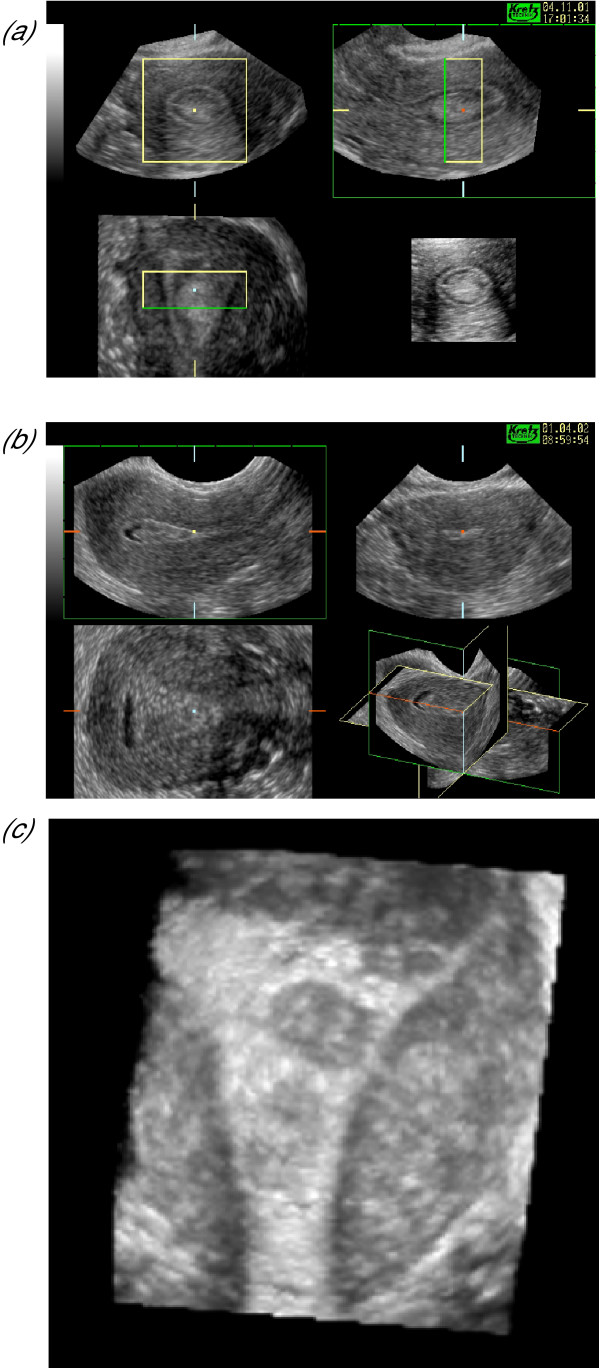
***Endometrial polyps***. Endometrial polyps are best appreciated during the late follicular phase when they can be seen against the plump heterogenous endometrium with its characteristic trilaminar pattern may be seen (Fig. 8a). Polyps may also be seen towards the end of menses however or at the 'down-regulation' scan when many women are still bleeding (see below) with the menstrual fluid acting as an in vivo contrast agent (Fig. 8b). The coronal view may be used to locate the position of intrauterine pathology and confirm the presence of individual smaller polyps suitable often confused as large single fibroids with conventional imaging that are suitable for routine polypectomy rather then hysteroscopic resection (Fig. 8c)

**Figure 9:**
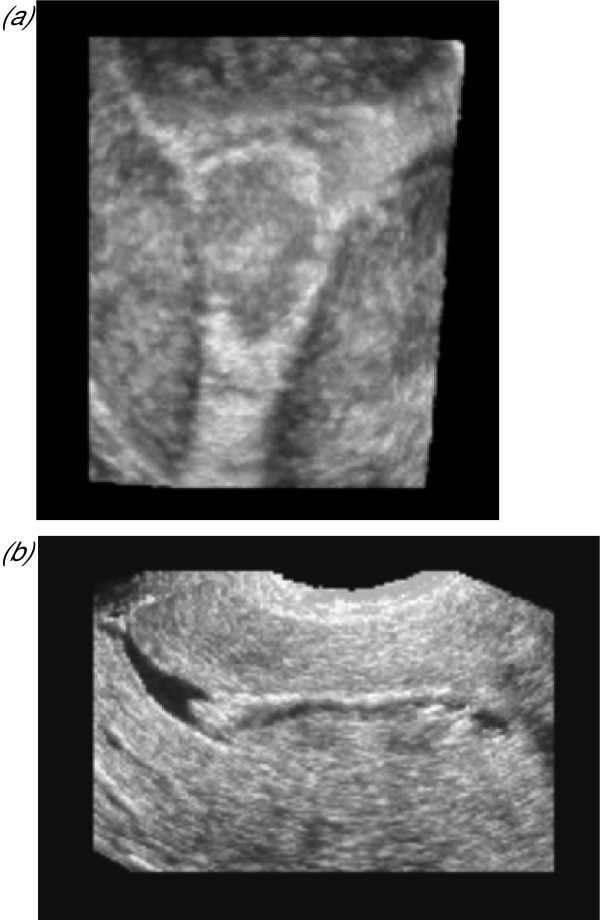
***Three-dimensional saline infusion contrast sonography***. Two-dimensional ultrasound had shown a persistently thick endometrium despite several weeks of pituitary suppression prior IVF treatment and saline infusion sonography has proven unhelpful simply confirming a thick endometrium despite very low serum oestradiol levels. A three-dimensional acquisition was undertaken therefore both before (Fig. 9a) and during the instillation of saline (Fig. 9b) and subsequently analysed off-line to reduce the amount of saline used and overall time needed for the examination. A large sub-mucosal leiomyoma with a broad base was seen originating from the left posterior aspect of the uterus that almost entirely occupied the cavity (Fig. 9b). This allowed the cancellation of the planned IVF treatment with the patient continuing her 'down-regulation' medication for a further two-months at which stage the fibroid was removed transcervically using a modified operative hysteroscope.

La Torre et al. compared three-dimensional sonography with conventional imaging with and without saline contrast in their study of twenty-three patients in whom subsequent hysteroscopy revealed the presence of 16 endometrial polyps [28]. Standard two-dimensional sonography demonstrated a relatively poor specificity of only 69.5% suggesting the presence of polyps in 23 patients. This was improved to 94.1% when two-dimensional sonography was used in conjunction with saline infusion as only 17 patients were then thought to have polyps. Three-dimensional sonography performed almost as well diagnosing the presence of polyps in 18 patients with a specificity of 88.8% and subsequently correctly identified all 16 polyps when used in conjunction with saline infusion. A similar improvement in specificity with three-dimensional sonography has been shown by Sylvestre et al. in their study of 209 subfertile patients thought to have an intrauterine lesion on transvaginal two-dimensional sonography or hysterosalpingography [29]. Using saline infusion sonography with two-dimensional and then three-dimensional sonography, 92 patients were subsequently identified as having a variety of intrauterine lesions suggesting a sensitivity and specificity of 97% and 11% for two-dimensional sonography, 87% and 45% for three-dimensional sonography and 98% and 100% for two-dimensional saline infusion sonography. Of 59 patients that had undergone hysteroscopy, the sensitivity and positive predictive value of saline infusion sonography were 98% and 95% when performed in combination with two-dimensional sonography and 100% and 92% with three-dimensional sonography respectively. The study clearly demonstrates how simple contrast media potentially increase the specificity of two-dimensional sonography as 55% (116 of 209) of patients were found to have normal cavities following the infusion of saline. This was largely due to the correct localization of leiomyomas as intramural rather than submucosal (54 of 101). This is extremely important in the clinical setting, as whilst three-dimensional sonography improves further on two-dimensional imaging it is not currently available in all units and its use therefore has significant implications in terms of cost and training. Saline infusion on the other hand may be undertaken easily using various dedicated systems or modifications of readily available cheaper catheters such as a paediatric feeding tube. Figure 8 shows the typical three-dimensional sonography appearances of endometrial polyps at different stages of the menstrual cycle whilst Figure 9 demonstrates a sub-mucous fibroid at three-dimensional saline infusion sonography.

#### • Tubal patency

The combination of contrast media and three-dimensional sonography has also been used to assess tubal patency. Kiyokawa et al. found three-dimensional saline sonohysterosalpingography was able to demonstrate the entire contour of the uterine cavity in 96% of cases compared to only 64% cases with conventional X-ray hysterosalpingography (p < 0.005) and was associated with a positive predictive value and specificity of predicting tubal patency of 100% in 25 unselected infertile patients [30]. Sladkevicius et al. found three-dimensional power Doppler imaging demonstrated free spill almost twice as often as conventional imaging (114 versus 58 tubes respectively) when used during hysterosalpingo-contrast sonography [31]. Sankpal et al. reported less promising results with the same technique when they reassessed tubal patency in 15 women who had normal X-ray HSG examinations within the previous year [32]. An important distinction was their use of saline rather than a positive contrast agent but it maybe that the technique has a distinct learning curve and numbers were small.

#### • Polycystic Ovaries

**Figure 10:**
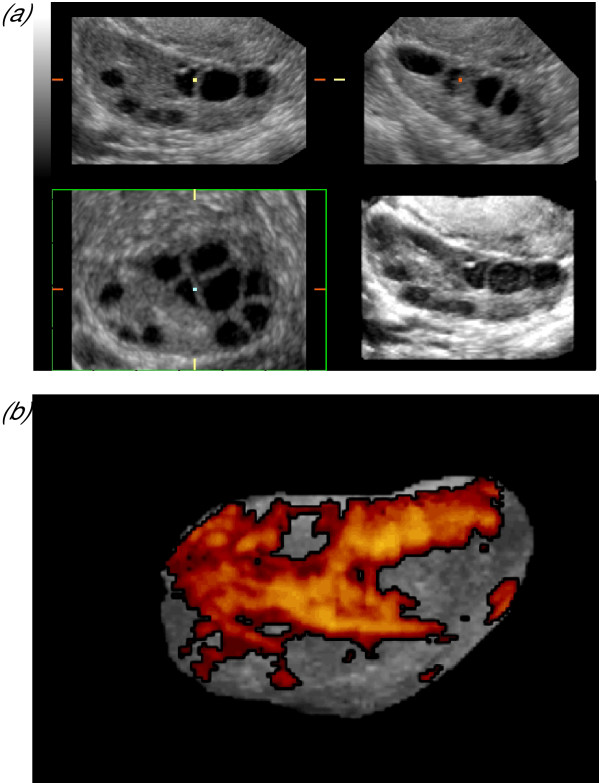
***Polycystic ovaries***. Three-dimensional sonography facilitates objective assessment of the ovarian stroma, through measurement of its mean grey signal intensity, its vascularity and its volume, which may be calculated by subtracting total follicular volume from total ovarian volume (Fig. 9A). Ovarian blood flow is increased and associated with significantly higher three-dimensional indices of vascularity than ovaries with a normal appearance (Fig. 10b).

Several groups have used three-dimensional sonography to demonstrate ovarian volume and vascularity are increased in polycystic ovarian syndrome [33,36]. Three-dimensional sonography also allows for the measurement of stromal volume through the calculation and subtraction of total follicular volume from total ovarian volume (Fig. 10). Using this technique Kyei-Mensah et al showed stromal volume was positively correlated with serum androstenedione concentrations (p < 0.01) in 26 women with clinical evidence of polycystic ovaries [37]. However, using a similar approach, Nardo et al. were unable to demonstrate any relationship between serum FSH, LH or testosterone and ovarian stromal volume in 23 infertile women with clomiphene citrate-resistant PCOS at the same stage of the menstrual cycle [38].

### Assisted reproduction treatment

Transvaginal sonography is used on a daily basis to monitor the response to treatment and to guide the transvaginal collection of oocytes and subsequent transcervical transfer of embryos to the uterus. Three-dimensional sonography may be used in any of these areas but has largely been applied as a predictor of ovarian response and as a determinant of endometrial receptivity. To clarify the current evidence in relation to the role of three-dimensional sonography in these areas it is necessary to first outline the general principles involved in assisted reproduction treatment.

#### • Principles of in vitro fertilisation therapy

The first stage is to obtain control over the hypothalamic-pituitary-ovarian axis through the induction of pituitary 'down-regulation' achieved through a continuous and highly supraphysiological dose of a gonadotrophin releasing hormone (GnRH) analogue. Follicular development is then induced through the administration of natural or synthetic Follicle Stimulating Hormone (FSH) until at least three large follicles measuring 18 mm or more are present when the resumption of meiosis is initiated though a single dose of human Chorionic Gonadotrophin (hCG), which has a similar structure and pharmacodynamic effect to Luteinising Hormone (LH) but is more readily available. Exposure to hCG is essential prior to the collection of the oocytes to ensure that they are mature and therefore capable of being fertilised when incubated with sperm during *in vitro *fertilisation (IVF) or when injected with a single sperm in intracytoplasmic sperm injection (ICSI) treatment. This would eventually lead to ovulation *in vivo *if the oocytes were not physically retrieved and this procedure is critically timed therefore to occur within 34 to 36 hours of the administration of hCG.

The dose and duration of FSH treatment is dependent upon each individual patients response to stimulation and is adjusted dynamically during treatment dependent on the number of follicles developing and on the amount of oestrogen they produce collectively as measured in the serum. The response and eventual number of mature oocytes retrieved is referred to as the patients ***'ovarian reserve'***. Prediction of ovarian reserve has obvious importance as it allows treatment to be tailored to the individual potentially increasing the number of oocytes retrieved.

Fertilisation of an oocyte is confirmed by the presence of two pronuclei 18 to 24 hours after IVF or ICSI. The embryos are allowed to develop *in vitro *until the second or third day after oocyte retrieval when most units would transfer a maximum of two to the recipient uterus selected on the basis of their quality as determined by a subjective grading system. The chance of the implantation occurring is not only dependent upon the embryo stage and grade but also on the endometrium. Just how important ***'endometrial receptivity' ***is in the overall process remains uncertain but the endometrium is likely to play more than a passive role.

With a better understanding of processes involved in controlled ovarian stimulation and assisted reproduction let us now turn to the application of three-dimensional sonography and critically review its current role.

#### • Three-dimensional markers of 'ovarian reserve'

Of the sonographic markers suggested as predictive of ovarian response the three that have been specifically addressed by three-dimensional sonographic studies are antral follicle counts, ovarian volume and ovarian blood flow.

**Figure 11:**
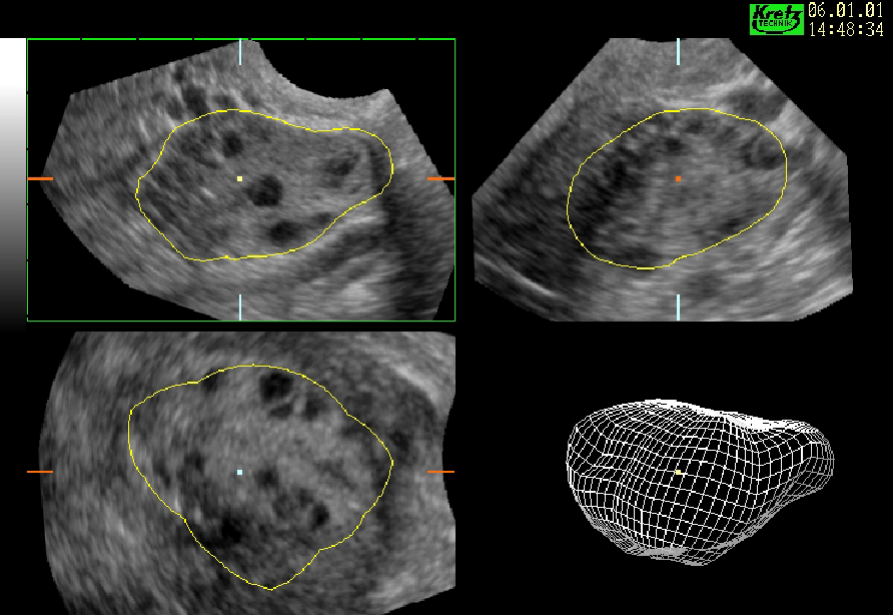
***Ovarian volume calculation***. Ovarian volume may be measured in the same way as endometrial volume through the manual delineation of the ovarian cortex.

**Figure 12:**
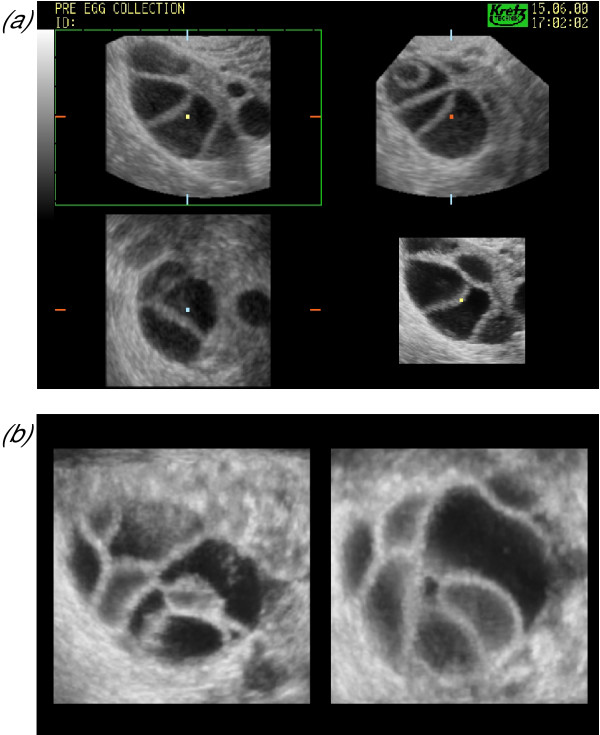
***Controlled ovarian stimulation***. Three-dimensional sonography may be used to facilitate standard measurement of follicular diameter **(a)**. During the latter stages of ovarian stimulation the ovaries may contain many follicles, which become progressively harder to measure reliably between observers, as there are no landmarks to aid orientation. Off-line analysis of stored three-dimensional datasets reduces the time spent with the patient and may potentially improve the number of mature oocytes retrieved by identifying the most appropriate time for oocyte collection. Evidence also suggests that three-dimensional rendering allows demonstration of the cumulus oophorus complex in follicles containing a mature oocyte **(b)**.

Pellicer et al. were amongst the first to use three-dimensional sonography as an adjunct to conventional markers of ovarian reserve when they examined ovarian volume and the number of 'selectable follicles' measuring 2–5 mm in a small group of low responders on day three of the menstrual cycle [39]. Both the number of selectable follicles and the total number of antral follicles were significantly decreased in the 'low responder' group who also demonstrated significantly higher serum FSH levels despite having values within the normal range. Ovarian volume measurements, however, were similar between the two groups. Pohl et al. also used three-dimensional sonography to quantify the number of follicles of varying diameter in 113 patients following 'down-regulation' but prior to ovarian stimulation ([40]. Patients with a higher number of follicles measuring between 5 and 10 mm were younger (p < 0.01), had a significantly higher number of oocytes retrieved (p < 0.0001) and were more likely to conceive (p < 0.05) (Fig. 12). Kupesic et al also suggest that the antral follicle count is a better predictor than three-dimensional measures of ovarian volume and blood flow [41]. A minimal ovarian volume may be important however (Fig. 11). Schild et al. noted a pregnancy rate of only 6.7% (1 of 15) in patients with a minimum unilateral ovarian volume of ≤ 3 cm^3^, which represented a single standard deviation below the mean, versus 21.9% (30 of 137) in patients with an initial minimum ovarian volume above 3 cm^3^[42]. This difference was not significant however and cancellation rates due to poor ovarian response or failed fertilisation were similar in both groups.

There is no doubt that antral follicle counts, when used in categorical classifications, are an important predictor of 'ovarian reserve' and may be measured with a high level of agreement both between and within observers [43]. Tree-dimensional sonography, however, does not appear to offer any significant advantage over two-dimensional imaging even at higher follicle counts when interobserver reliability is reduced. Ovarian volume has a limited predictive ability that does not appear to supersede that of antral follicle counts. Do measurements of ovarian vascularity add anything to validate the use of three-dimensional sonography as a marker of ovarian reserve?

Jarvela et al. used three-dimensional power Doppler angiography after pituitary 'down-regulation' and during gonadotrophin stimulation to compare ovarian vascularity in 33 women with normal ovarian reserve, as judged by antral follicle counts, to 12 women who had demonstrated a previous poor response [44]. The number of oocytes retrieved correlated with the antral follicle count (R = 0.458, p < 0.01) and ovarian volume (R = 0.388, p < 0.05) but not with ovarian vascularity. All three indices of vascularity were shown to increase significantly during gonadotrophin stimulation in the group with normal ovarian reserve only but this was related to the antral follicle count reiterating the importance of this marker as an independent variable. Kupesic et al. similarly showed the number of oocytes retrieved and subsequent conception rate to be greater in patients with a greater ovarian volume and a greater ovarian stromal vascularity but not independently of a higher number of antral follicles [45].

#### • Three-dimensional markers of 'endometrial receptivity'

Schild et al. measured endometrial thickness and volume in a total of 47 IVF cycles on the day of oocyte retrieval [46]. There were no significant differences between the group of fifteen patients that conceived (31.9%) and the remaining 32 non-pregnant women in terms of the mean endometrial thickness (10.8 ± 2.3 mm versus 11.8 ± 3.4 mm) or volume (4.9 ± 2.2 cm^3 ^versus 5.8 ± 3.4 cm^3^) respectively. Yaman et al. reported similar findings with no differences in endometrium volume (4.16 ± 1.97 cm^3 ^versus 4.53 ± 1.79 cm^3^), endometrium thickness (11 ± 2 mm versus 11 ± 2 mm) between 21 pregnant and 44 non-pregnant women on the day of hCG administration [47]. However, whilst there was no absolute endometrial thickness required for pregnancy a minimal endometrial volume above 2.5 cm^3 ^favoured pregnancy. Raga et al. also described a cut-off point in endometrial volume in their study of 72 patients on the day of embryo transfer [48]. Implantation rates were significantly lower (p < 0.05) in those women with endometria measuring less than 2 cm^3 ^and no pregnancies were observed at volumes below 1 cm^3^. Even if there is a lower limit in endometrial volume below which pregnancy will not occur conventional measurement of endometrial thickness is already known to have a similar negative predictive value with conception less likely in patients with an endometrium measuring less than 5 mm in diameter.

Kupesic et al. reported more predictive information could be derived at the time of embryo transfer when three-dimensional power Doppler was used to quantify endometrial vascularity [49]. Of 89 patients studied successful conception cycles were associated with a significantly higher endometrial flow index (13.2 ± 2.2 versus 11.9 ± 2.4, p < 0.05). Wu et al. also found three-dimensional power Doppler angiography to be an important determinant of 'endometrial receptivity' but on the day of hCG administration in 54 patients undergoing their first IVF cycle [50]. The subendometrial vascularisation flow index (VFI) proved the best predictor of conception being superior to the vascularisation index (VI), flow index (FI) and endometrial volume in the receiver operating characteristics curve analysis. Interestingly, three-dimensional sonography may also be used to examine endometrial vascularity and determine 'endometrial receptivity' prior to ovarian stimulation. Schild et al. reported significantly lower indices of vascularity at down-regulation in 15 patients that subsequently conceived (20%) than in 60 non-conception cycles (p < 0.05) with the flow index the strongest predictive factor of IVF success (p < 0.05) [51]. Endometrial measurements were once again not correlated with outcome. This may reflect a more profound pituitary suppression but is more likely to reflect patients responsive to exogenous hormonal therapy.

### Applications in early pregnancy

#### • Miscarriage

Gestational sac volume has been proposed as representative of the competence of the early uteroplacental unit and therefore a potential predictor of pregnancy outcome [52]. In one of the first reported applications of three-dimensional sonography, Steiner et al. studied 38 pregnancies between 5 and 11 weeks gestation and found gestational sac volume measurements to significantly correlated with gestational age (r = 0.74, p < 0.001) and more than two standard deviations below the mean in three of five embryonic or anembryonic pregnancy failures [52]. However, Acharya and Morgan have recently shown a clear relationship between gestational sac volume and sac diameter also exists in patients with embryonic pregnancy failure and that measurements are unable to predict eventual outcome during conservative management [53]. Babinszki et al. did find a relationship between gestational sac and yolk sac volume and adverse outcome in a group of 49 patients who had conceived following treatment for subfertility but crown rump length performed equally as well [54].

Currently, therefore, one may conclude that whilst there is a distinct relationship between gestational age and three-dimensional measurements of gestational and yolk sac volume, these parameters do not appear to improve upon the predictive value of current tests in determining the eventual outcome in either viable or non-viable intrauterine pregnancies.

#### • Ectopic pregnancy

**Figure 13:**
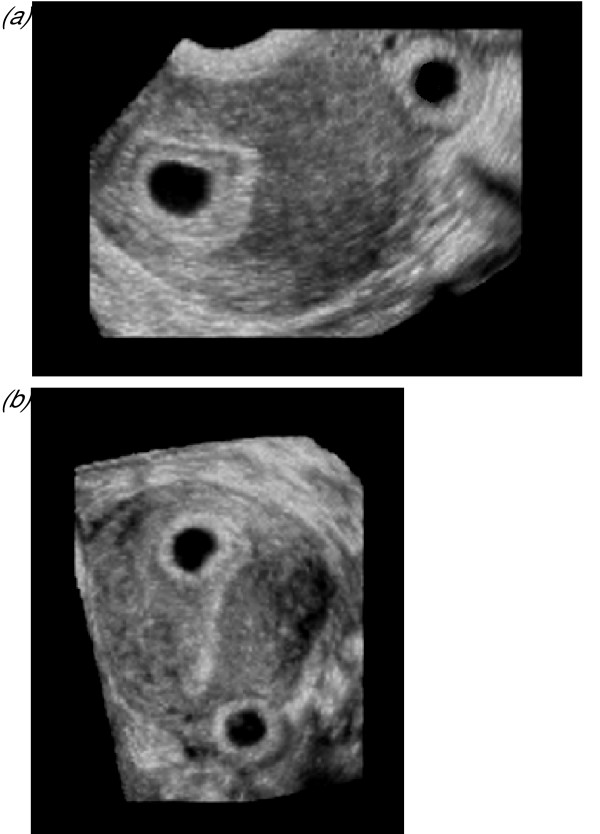
***Ectopic pregnancy***. The spatial orientation of three-dimensional ultrasound and its ability to demonstrate the coronal plane of the uterus in its natural position may help in the diagnosis and exclusion of ectopic pregnancy. This is particularly true in cases of heterotropic pregnancy where an intrauterine pregnancy is associated with an ectopic twin gestation. Spontaneous heterotropic pregnancy is rare but may complicate 0.5% of pregnancies arising from IVF treatment with the ectopic gestation located in the cornua (Fig. 13a) or cervix (Fig. 13b).

The improved spatial orientation afforded by three-dimensional sonography has been used to evaluate endometrial shape in cases of pregnancy of unknown location in an attempt to improve the sensitivity and specificity of sonography. Rempen et al. described the persistence of a distinct symmetry with regard to the median longitudinal axis of the uterus in the coronal plane in 90% of extrauterine pregnancies which was lost in intrauterine pregnancies [55]. Su et al. reported a case of an anembryonic cervical pregnancy diagnosed at 10 weeks associated with a large arteriovenous malformation managed conservatively with selective uterine artery embolisation [56]. Three-dimensional power Doppler ultrasonography proved useful in the initial diagnosis subsequent monitoring of the response to treatment. These studies suggest a potential role for three-dimensional sonography but in the absence of properly blinded data it is impossible to predict whether it will improve upon conventional sonography. The spatial orientation and additional information afforded by the coronal plane do lend themselves to assessment of the endometrial cavity as illustrated so elegantly by the three-dimensional studies on uterine anomalies so one should be optimistic in this regard. Certainly in our own unit we have found three-dimensional imaging to be beneficial in several cases of pregnancy of unknown location and particularly so in twin pregnancies following assisted reproduction treatment and embryo transfer where heterotropic pregnancy is so much more likely (Fig. 13).

## Conclusion

There is sufficient evidence to support the notion that the theoretical advantages of three-dimensional sonography are indeed translated into clinical practice in the field of reproductive medicine. The spatial orientation and additional information derivable from individual sectional planes has greatly enhanced our knowledge of uterine anomalies and contributed to our understanding of how these affect pregnancy outcome and may offer insight into the location of pregnancies of unknown location. Quantitative three-dimensional analysis of volume and vascularity has proven less powerful and whilst individual studies suggest a potential role for such measurements these do not appear to out perform current assessments.

For now three-dimensional sonography largely remains an exciting research tool with the converted applying it in different forms and areas of interest and in doing so unearthing significant new information about normal physiology and pathophysiological change that direct further work. Three-dimensional sonography offers too much to be ignored and, as history has shown with previous developments, will gradually become commonplace in most units. Our role is to continue to test it prospectively but to remain realistic and to examine how it may be most appropriately applied in the clinical setting. Be a pioneer, embrace it and you will be rewarded handsomely!
